# Evaluation of classification in single cell atac-seq data with machine learning methods

**DOI:** 10.1186/s12859-022-04774-z

**Published:** 2022-09-21

**Authors:** Hongzhe Guo, Zhongbo Yang, Tao Jiang, Shiqi Liu, Yadong Wang, Zhe Cui

**Affiliations:** Faculty of Computing, Harbin Institute of Technology, Harbin, 150001 China

**Keywords:** scATAC-seq, Machine learning, Cell-type classification, Evaluation, SVM

## Abstract

**Background:**

The technologies advances of single-cell Assay for Transposase Accessible Chromatin using sequencing (scATAC-seq) allowed to generate thousands of single cells in a relatively easy and economic manner and it is rapidly advancing the understanding of the cellular composition of complex organisms and tissues. The data structure and feature in scRNA-seq is similar to that in scATAC-seq, therefore, it’s encouraged to identify and classify the cell types in scATAC-seq through traditional supervised machine learning methods, which are proved reliable in scRNA-seq datasets.

**Results:**

In this study, we evaluated the classification performance of 6 well-known machine learning methods on scATAC-seq. A total of 4 public scATAC-seq datasets vary in tissues, sizes and technologies were applied to the evaluation of the performance of the methods. We assessed these methods using a 5-folds cross validation experiment, called intra-dataset experiment, based on recall, precision and the percentage of correctly predicted cells. The results show that these methods performed well in some specific types of the cell in a specific scATAC-seq dataset, while the overall performance is not as well as that in scRNA-seq analysis. In addition, we evaluated the classification performance of these methods by training and predicting in different datasets generated from same sample, called inter-datasets experiments, which may help us to assess the performance of these methods in more realistic scenarios.

**Conclusions:**

Both in intra-dataset and in inter-dataset experiment, SVM and NMC are overall outperformed others across all 4 datasets. Thus, we recommend researchers to use SVM and NMC as the underlying classifier when developing an automatic cell-type classification method for scATAC-seq.

## Background

In recent years, scATAC-seq provides the opportunity to accurately and sensitively identify and characterize the cells in complex tissues. scATAC technologies enable profiling the epigenetic landscapes of thousands of individual cells in a relatively easy and cost-effective way, resulting in the development of computational methods to analyze and interpret data. Due to the similarity of data structure and feature selection between scRNA-seq and scATAC-seq data, the procedures for analyzing the composition of cells in complex tissues from scRNA-seq [1] can also apply in scATAC-seq dataset. To be more specific, the analysis of a scATAC-seq dataset typically start by unsupervised clustering of cells based on the peaks feature in chromatin accessibility profile, and then followed by the clustered group annotation by assigning a label to each cell based on specific markers. These steps are convinced and valuable in identifying and characterizing various cell types by extensive results carried out before, which has resulted in the atlas of chromatin accessibility in mouse [2]. However, the traditional annotation approach mentioned above is still deficient with wasting a great large amount of time on literature review and manual inspection of cluster-specific marker. With the huge increase of cell population size and sample size, such labor-intensive shortage on cell-types annotation become more fatal than before, preventing accurate, sensitive and efficient cell identification.

In order to overcome such challenges in cell-type annotation, a plenty of classification methods including support vector machine (SVM) [3, 4], neural network (NN) [5, 6], Linear Discriminant Analysis (LDA) [7], random forest (RF) [8–10], k-Nearest Neighbors (KNN) [11] and nearest mean classifier (NMC) [11] have been developed as underlying algorithm for automatically labeling cells in scRNA-seq datasets. A recent result [12] showed that SVM [13] performs best on the cell-type classification in several scRNA-seq datasets vary in species, sizes and technologies. Meanwhile, other traditional machine learning methods also perform as good as the mainstream tools on some specific datasets and scenarios. Hence, we anticipate that those machine learning methods proved convincing and valuable in scRNA-seq can also perform well in scATAC-seq thanks to the similarity in data structure and peak feature selection between scRNA-seq and scATAC-seq. By far, there is still no practical guidance for those machine learning methods available and lacking studies that comprehensively compare their performance on scATAC-seq. Obviously, the assessment result of these machine learning methods can both benefit the future developing of tools in this filed and provide significant guidance on the choice of methods for improving its performance and thus prevent unnecessary complexity.

**Fig. 1:**
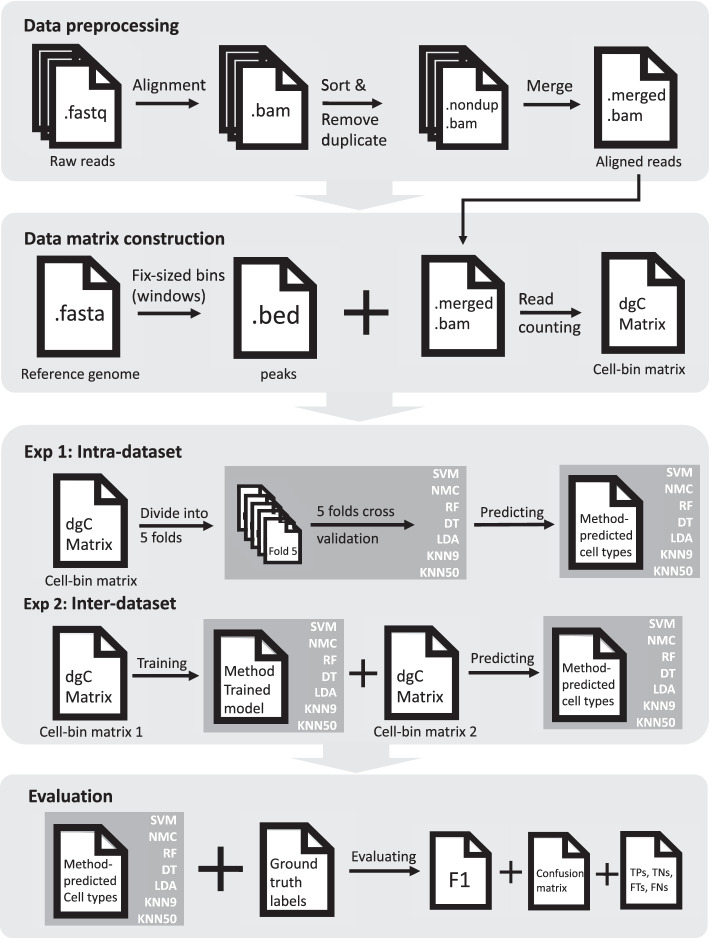
Schematic overview of single-cell ATAC-seq. In data preprocessing step, raw sequencing data in.fastq format for each cell will be aligned first and stored in.bam format. Then we will sort bam files and remove duplicate reads in each cell. Finally we will integrate all files together to a merged and sorted bam file. In order to construct a cell-bin matrix, we will first call a fix-sized bins (window) list, then based on this peak file and bam file to construct a cell-bin matrix stored in dcCMatrix class. Both experiment will output predicted cell types for each method. These predicted cell types will be evaluated with ground truth cell types at the end in several aspect, including F1 score, confusion matrix, etc

Here, we evaluated 6 popular machine learning methods (SVM, KNN, RF, LDA, NMC and Decision tree (DT)) to automatically assign cell labels in scATAC-seq datasets. These 6 machine learning methods were evaluated using 4 public available scATAC-seq datasets (Detailed in Table 1) including data of human immune cell (hereafter Corces2016) [14], data of human hematopoietic system cell (hereafter Buenrostro2018) [15] and data of peripheral blood mononuclear cell from the same healthy donor but prepared in two different libraries (hereafter 10 × PBMCs v1 and 10 × PBMCs Next Gem). Based on the 4 scATAC-seq datasets above, we evaluate the classification performance of these 6 methods through two steps: (1) intra-dataset experiments in which we applied a fivefold cross validation on each dataset and (2) inter-dataset experiments in which we compare the prediction ability of each method between 10 × PBMCs v1 and 10 × PBMCs Next Gem (Detailed in Fig. 1). Compared to the intra-dataset experiment for testing the classification ability of each method on a single dataset, inter-dataset experiment is much more practical and realistic as for each machine learning method, a reference dataset will be trained, and then its trained model will be applied to predicte cell types in a different unannotated datasets with same cell-type composition. Since there are only a small number of labeled scATAC-seq datasets available at this time, we applied 10 × PBMCs v1 data and 10 × PBMCs Next Gem data, which are generated in different prepared libraries and technologies from the same healthy donor are applied in the inter-dataset experiments, to evaluate the performance of each method on precision and efficiency identifying the cell types. For intra-dataset experiment, SVM and NMC both showed the competence of predicting the cell types in complex issues with satisfactory performance than other four machine learning methods. For inter-dataset experiments, SVM overall is the best performing one in all these supervised machine learning methods, which is consistent with results in scRNA-seq. SVM is also the most potential methods among all the supervised machine learning methods mentioned above, which means the SVM-based development of cell-type classification tools may be more efficient and accurate.

**Table 1:** Overview of datasets used in this study

Dataset	No. of cells	No. of populations	Description	Protocol	Source
Corces2016	575	4	Human immune cell	Illumina NextSeq 500	[14]
Buenrostro2018	2034	10	Human hematopoietic system cell	Illumina NextSeq 500	[15]
10 × PBMCs v1	3917(2927 labeled)	7	Peripheral blood mononuclear cell	10 × Chromium Next GEM Single Cell ATAC Reagent Kits v1.1	https://support.10xgenomics.com/single-cell-atac/datasets/1.2.0/atac_pbmc_5k_nextgem
10 × PBMCs Next Gem	4585(3670 labeled)	7	Peripheral blood mononuclear cell	10 × Chromium Single Cell ATAC Reagent Kits	https://support.10xgenomics.com/single-cell-atac/datasets/1.2.0/atac_pbmc_5k_v1

## Results

### SVM performs best on intra-dataset experiments across all datasets

**Fig. 2:**
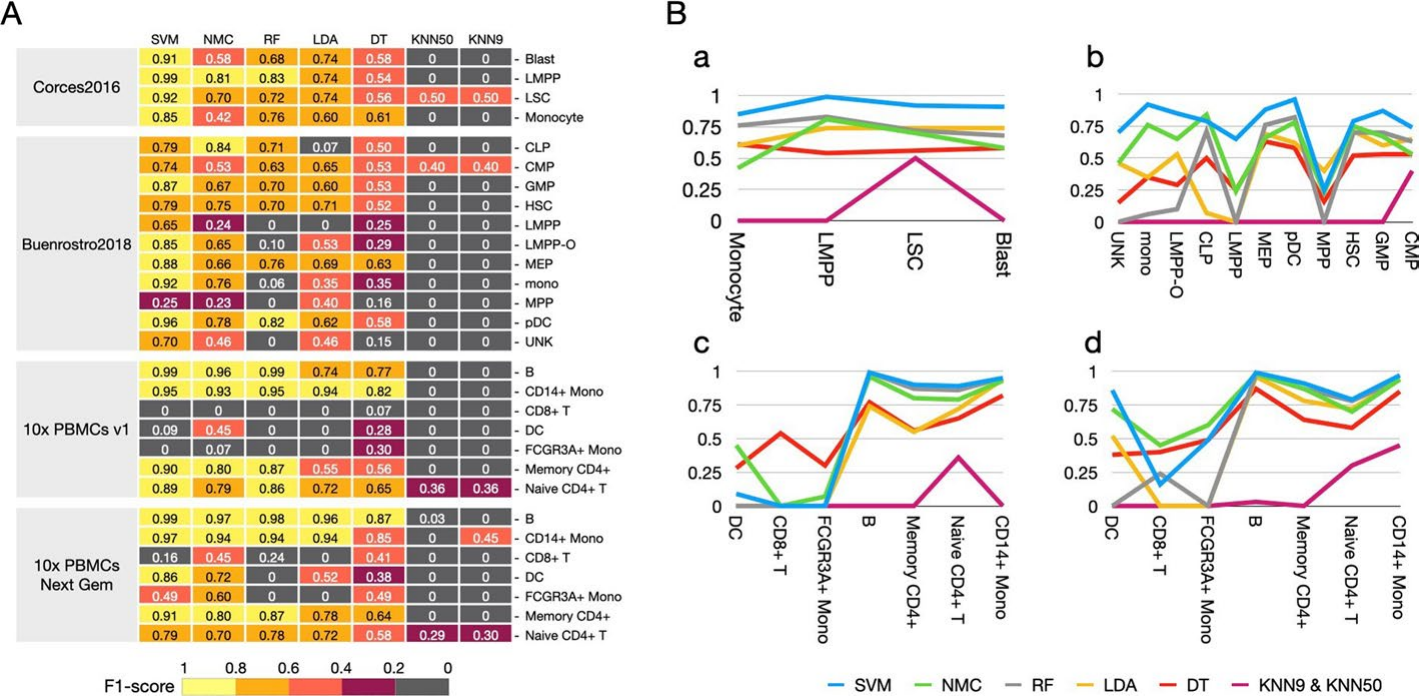
**A.** Heat map displaying the performance comparison of machine learning methods for cell identification using different scATAC-seq datasets. Methods are ordered based on the mean of the F1-scores. **B.** Line plot displaying the performance comparison of machine learning methods for cell identification using different scATAC-seq datasets. The line plot of F1 score for a) Corces2016. b) Buenrostro2018. c) 10 × PBMCs v1. d) 10 × PBMCs Next Gem. Cell type in each method are ordered based on the population size. KNN9 and KNN50 are merged and displayed together

We have compared the 6 methods based on their ability to profile and identify the various cell types. Generally, SVM performed best among all machine learning methods in intra-dataset experiments across most cell types in various datasets (Fig. 2). In contrast, KNN no matter with setting 9 or 50 nearest neighbors performed poorly in all datasets with only a few cells are correctly characterized.

Corces2016 dataset consisting only four human immune cell types (Blast, LMPP, LSC and Monocyte) which make the identification and classification easier than other datasets for all the machine learning methods. For Corces2016 dataset, SVM performed best in all 4 cell types. SVM as the top performing scATAC-seq data analysis method had the F1 score of all cell types surpassed 0.85, followed by RF and LDA with the average of F1 obtained 0.75 and 0.79 respectively. However, both KNN with setting 9 nearest neighbors (KNN9) and KNN with setting 50 nearest neighbors (KNN50), only got F1 about 0.50 in LSC, performed worst across all four cell types (more details in Fig. 2A). The performance on other three datasets showed large similarities with the Corces2016, but had a little bit difference. SVM still performed best in Buenrostro2018 dataset. For 10 × PBMCs v1 dataset, SVM got best performance on 4 cell types including B, CD14 + Mono, Memory CD4 + and Naive CD4 + T For 10 × PBMCs Next Gem dataset, SVM got the best performance on 5 cell types including B, CD14 + Mono, DC, Memory CD4 + and Naive CD4 + T. Though overall SVM still showed the best performance among all the 4 datasets, some methods worked better in specific cell types under different datasets. To be more specific, LDA got a better F1 score on MPP in Buenrostro2018. NMC got a better F1 score on DC and FCGR3A + Mono, and DT got a better F1 score on CD8 + T, DC and FCGR3A + Mono in 10 × PBMCs v1. In 10 × PBMCs Next Gem, NMC performed better on CD8 + T and FCGR3A + Mono, which may be caused by the different underlying biology feature and population. Unfortunately, KNN9 and KNN50 still didn’t competent enough to profile the cell types sensitively and accurately. They only had unsatisfactory F1 under 0.45 on CMP in Buenrostro2018, F1 less than 0.36 and 0.30 on Naïve CD4 + T in 10 × PBMCs Next Gem and 10 × PBMCs Next Gem. In spite of this, KNN9 and KNN50 were still the worst methods in these three datasets.

### The classification performance of methods does not depend on population size

Generally, a cell type with larger population number may contains more unique characteristics, which benefit the classifier to separate discrete cell type from others in complex issues and organisms. On the contrary, the large number of cells may increase the complexity of feature, make it hard to classify the cell types with high performance. We tried to find the dependence between the classification performance of methods and the cell population size. Unfortunately, in intra-dataset experiment, we did not find a strict consistency between the performance of classifiers and population size in all the four datasets (Fig. 2B). Because of the relatively small population about 575 cells in Corces2016 and its four cell groups size (Monocyte, LMPP, LSC and Blast) is relatively close, almost all machine learning methods had better performance on all the cell types. In Buenrostro2018 dataset, though there are two decreases on LMPP and MPP, these methods overall work well along with the cell numbers compared to the 10 × PBMCs v1 and 10 × PBMCs Next Gem datasets. The trend of F1 scores in 10 × PBMCs v1 is consistent with that in 10 × PBMCs Next Gem dataset, that is, all the classifiers performed relatively bad on DC, CD8 + T and FCGR3A + Mono cells and performed better on other cell groups, except KNN9 and KNN50 performing bad in all cell types, which indicates that this trend is caused by the smaller cell groups with less character had more complexity in classification. Though there is no strict consistency between the population size and the performance of method in all datasets, the larger number of cells generally performed well at a relatively higher level, compared to the performance of smaller cell population.

### Performance evaluation across datasets (inter-dataset experiment)

**Fig. 3:**
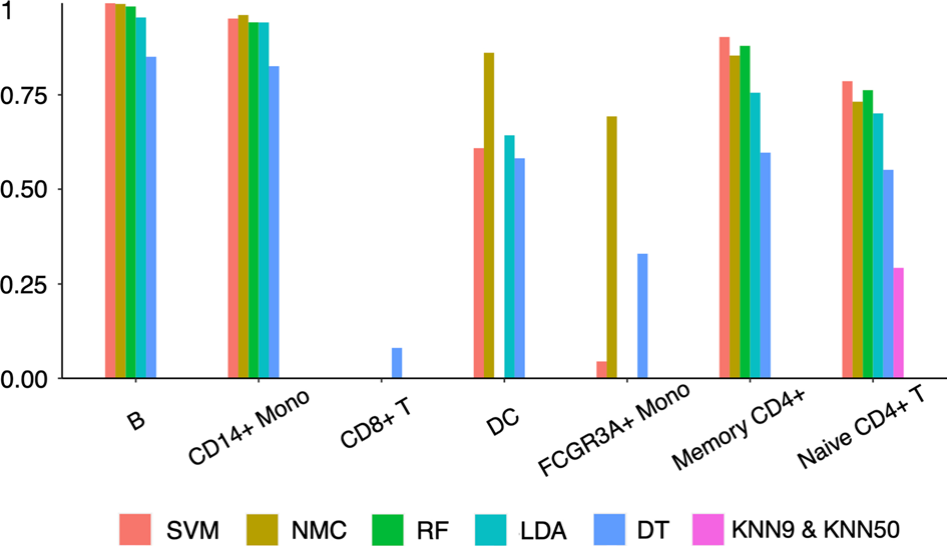
Bar plot for F1 score comparing the performance of each method in each cell type. Cell types are ordered based on the population size (from small to large). SVM and NMC got highest F1 score on these cell types, and KNN9 and KNN50 got the only F1 score on the last cell type. NMC got the highest mean F1 score of 0.73 while KNN9 and KNN50 got the lowest score of 0.04

**Fig. 4:**
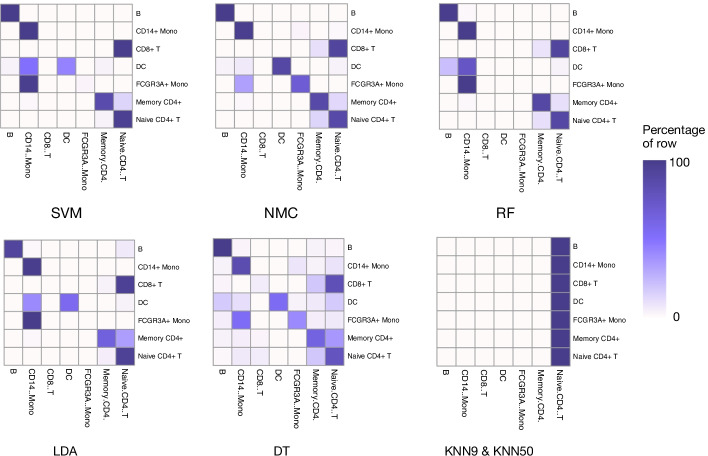
Performance comparison of machine learning methods for cell identification using different scATAC-seq datasets. Heat map comparing the predicted cells versus true label of each method including SVM, NMC, RF, LDA, DT and KNN9&KNN50. Color represents the percentage of cells of a certain reported type labeled as each type

**Fig. 5:**
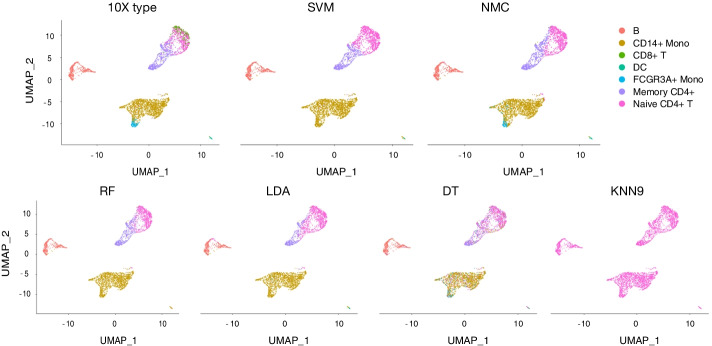
UMAP visualization of each method for performance comparison in intra-dataset experiment. The first panel is colored by cell type based on the golden standard of 10 × PBMCs Next Gem dataset (Seurat v3 labeled), the second to seventh panel is colored by labeled cell type according to SVM, NMC, RF, LDA, DT and KNN9 & KNN50, respectively

Intra-dataset, evaluating the classification performance within a dataset, is important to figure out the ability of these machine learning methods to discriminate the cell types. In addition, the realistic scenario in which a method is useful requires cross-dataset (i.e., inter-dataset) classification is essential in the evaluation. We applied these machine learning methods to 10 × PBMCs v1 and 10 × PBMCs Next Gem datasets from a same donor but profiled to a different molecular depth with a different library preparation method and different chromium system (v1 chemistry and Next Gem v1.1 chemistry) into the inter-dataset experiment. In detail, we first trained the model using 10 × PBMCs v1 dataset and then test the classification ability on 10 × PBMCs Next Gem dataset to get the methods that have the stronger ability to predict the cell types with higher performance. All experiment results are summarized in Figs. 3, 4 and 5. Overall, SVM and NMC are the 2 best-performing machine learning methods among other classifiers in this experiment with the average F1-score at 0.61 and 0.73 (Fig. 3). SVM and LDA performed best on B, CD14 + Mono, DC, Memory CD4 + and Naive CD4 + T these 5 cell types. NMC performed well on 6 cell types (B, CD14 + Mono, DC, FCGR3A + Mono Memory CD4 + and Naive CD4 + T). While DT is the best and only one method than could work on CD8 + T cell types though the performance was not satisfactory enough. Not surprisingly, KNN9 and KNN50 still performed worst with identifying all cells into one group (details in Fig. 4).

In this experiment, most of machine learning methods had competent ability to are distinguish B, Memory CD4 + and Naive CD4 + T cell types from each other correctly. However, only NMC, DT and SVM successfully separated part of FCGR3A + Mono from CD14 + Mono, while others mistakenly mix these two cells together (Fig. 5). Similarly, almost all methods failed to separate CD8 + T cells from Naive CD4 + T cell group, while only DT correctly identified a small number of CD8 + T cells. Additionally, most of machine learning methods mis-identified FCGR3A + Mono to CD14 + Mono. Despite the performance of these machine learning methods varied in inter-dataset experiment, the overall performance of these classifiers was relatively worse by comparing to the F1 scores and percentage of cells of each cell type in scRNA-seq dataset. The results figured out provided important guidelines for the further development of the scATAC-seq machine learning methods.

## Discussion

Taken together, we evaluated the performance of 6 current state-of-art machine learning methods for identify and classify the cell types automatically using 4 available scATAC-seq datasets. In this study, we designed 2 testing scenarios, that is, an intra-dataset and an inter-dataset experiment, to test the ability to distinguish and profile the cell types of traditional machine learning methods in scATAC-seq comprehensively. Overall the 4 datasets, several traditional methods including SVM, NMC and RF could distinguish the various cell types with relatively high performance in intra-dataset experiment. In contrary, compared to the intra-dataset experiment, we observed relatively worse performance for the inter-dataset experiment, likely due to the technical and biological differences between the datasets. SVM and NMC outperformed among the other methods both in intra-dataset and inter-dataset experiment, however, KNN9 and KNN50 performed worst for the both experiments. It’s worth noting that, although adjusting the settings for a specific dataset might improve the performances, it increases the risk of overtraining. In order to reduce the risk, we evaluated all methods using the default settings except setting 9 and 50 nearest neighbors for KNN9 and KNN50 used to identify smaller and larger population. Moreover, we observed that there is no significant consistent trend between the population size of cell and the accuracy of classification. In another word, the performance of the methods didn’t depend on the population of the cell types.

In the inter-dataset experiments, we tested the performance of the methods to classify the populations using the trained model across different scATAC-seq protocols. Due to the limitation of available dataset, two 10 × PBMCs datasets which are sampled from a same healthy donor but sequenced with different prepared libraries and different chromium systems were applied to the inter-dataset experiment. The results showed that these two datasets are compatible, SVM and NMC outperformed than other 4 machine learning methods in 5 cell groups. Unfortunately, KNN9 and KNN50 are still the performed worst with barely correctly identifying and identifying all cells types into one group.

These results play an important role for research to choose the most high-performance traditional machine learning method for the analysis of scATAC-seq data as underlying classifier when researcher try to develop an automatic classification method based on scATAC-seq.

## Conclusions

All in Although some methods performed well in the datasets we chose, the existing machine learning methods couldn’t produce convincing results for developing a scATAC-seq automatic classification method, thus they still cannot be used directly as underlying classifier. In order to obtain higher performance, we encourage that researcher to:

- As far as possible to choose a machine learning method which is most compatible for the specific dataset including the biological problem and biological problem. SVM and NMC may performed better than other traditional method for general datasets. However, the default parameters and settings we used in these methods may not reach the best performance in specific datasets, thus the developer need to adapt and adjust accordingly.
- A modification to the kernel function or a correction and integration to the read signal in scATAC-seq data is essential and important for the accuracy and sensitivity of following classification. As mentioned before, there is a great loss in read signal because of the sequencing technique, we encourage the research to recover or enhance the read signal before training and predicting, which may enhance the recognition of features.

## Methods

### Machine learning methods

We evaluated 6 state-of-art machine learning methods on different scATAC-seq datasets, publicly available as Python packages (Fig. 1). These 6 traditional methods are all from the scikit-learn library in Python [13]: support vector machine (SVM) with linear kernel, nearest mean classifier (NMC), random forest (RF), decision tree (DT), linear discriminant analysis (LDA) and k-nearest neighbor (KNN). Specially, for KNN, both 9 neighbors and 50 neighbors were chosen. For 9 neighbors, KNN could predict relatively small populations with good performance, and for 50 neighbors, it may identify the cell types sensitive and accurate on large population. After filtering the scATAC-seq datasets, cell populations consisting of at least 10 cells would be remained to the further experiment.

### Datasets

Corces2016, Buenrostro2018, 10 × PBMCs v1 and 10 × PBMCs Next Gem, these 4 datasets were used to assess all the 6 machine learning identification methods, from which all the 4 datasets were apply for the intra-dataset experiment using a fivefold cross-validation scheme, and the 10 × PBMCs v1 and 10 × PBMCs Next Gem datasets were used for further inter-dataset experiment. The datasets we used are various both in different tissue (Human immune cell, Human hematopoietic system cell and PBMC), size (from 575 to 4585) and the sequencing protocol (Illumina NextSeq 500 and 10 × Chromium). The human immune cell (Corce2016) is available from the NCBI database (GSE74310). The peak and count file of human hematopoietic system cell could be downloaded at GSE96769.The 10 × PBMCs v1 dataset and 10 × PBMCs Next Gem dataset were obtained from 10 × website (Table 1). These two 10 × PBMCs datasets are downloaded with only cluster group ID available, then we assigned label to each cell by Seurat v3 [16] since it can assign labels for scATAC-seq data with high convincement when its scRNA-seq and labels are available (Detailed in “Assign label for 10 × PBMCs data” section). The scRNA-seq and labels used for Seurat v3 is available from https://support.10xgenomics.com/single-cell-gene-expression/datasets/3.0.2/5k_pbmc_v3.

### Data preprocessing

For those raw sequencing data, we first aligned it to hg19 using BWA-MEM (version 0.7.17-r1188) [17] and then remove the replicate reads through Picard [18] and Samtools (version 1.9) [19]. Based on the aligned data and cell labels provided in the datasets, we then bin the full genome into fixed size windows (5 kb) and estimated read coverage for each bin to build a bins-by-cells binary count matrix. Bins that overlap ENCODE-defined blacklist regions are all set to zero. Cell population only more than 10 will be remained and less than 10 will be filtered. Note that no column that all values are 0 is filtered, which could be a kind of feature for a machine learning classifier. Each cell matrix is displayed in a compressed, sparse, column-oriented numeric matrix, in which a column represents a peak feature and a row represents a cell. These matrices are all stored in RDS files.

### Assign labels for 10 × PBMCs data

For 10 × PBMCs v1 and 10 × PBMCs Next Gem data, no label is provided for each cell when the datasets were first downloaded from website. Based on the labeled scRNA-seq data of the same healthy donor, we then predict the labels for scATAC-seq data using a Seurat v3, which is fast and can accurately detect the true biology connection, to select the high confidence labels as golden standard. Using this process, we totally labeled 2927 of 3917 cells and 3670 of 4585 cells for v1 dataset and Next Gem dataset respectively. Finally, only the labeled cells were kept for downstream analysis after filtered the 10 × PBMCs v1 and 10 × PBMCs Next Gem dataset.

### Intra-dataset & Inter-dataset classification experiment

For intra-dataset experiment, we evaluated the performance by applying a fivefold cross-validation across each dataset for those supervised machine learning methods. The folds were divided in a stratified manner in order to ensure equal proportions of each cell population in each fold. The training and testing folds were exactly the same for all methods. A total of 28 experiments (7 methods and 4 datasets) were applied for all methods.

For inter-dataset experiment, we used the 10 × PBMCs v1 and 10 × PBMCs Next Gem datasets to test the ability of machine learning methods in realistic scenarios. We trained a classifier using 10 × PBMCs v1 data and then test the performance of identifying various cell types on 10 × PBMCs Next Gem dataset. A total of 7 experiments were applied for all methods.

## Data Availability

All data generated or analysed during this study are included in this published article and its supplementary information files. Source code is available at https://github.com/mrcuizhe/scATAC-MachineLearning-benchmarking.
